# Quantitative non-nutritive sucking measurement as a predictor of oral feeding readiness in newborns

**DOI:** 10.3389/fped.2023.1143416

**Published:** 2023-08-11

**Authors:** Kyeong Jae Lee, Yong Beom Shin, Ho Eun Park, Suro Choi, Jean G. de Oliveira, Daun Hong, Sohee Kim, Jin A Yoon

**Affiliations:** ^1^ Department of Robotics and Mechatronics Engineering, Daegu Gyeongbuk Institute of Science and Technology (DGIST), Daegu, Republic of Korea; ^2^ Department of Rehabilitation Medicine, Pusan National University School of Medicine and Biomedical Research Institute, Pusan National University Hospital, Busan, Republic of Korea; ^3^ School of Undergraduate Studies, DGIST, Daegu, Republic of Korea

**Keywords:** oral feeding, silver nanowire-based flexible pressure sensor, nutritive sucking, preterm, non nutritive sucking

## Abstract

**Background and purpose:**

The purpose of this study is to examine the relationship between the parameters of a silver nanowire-based flexible pressure sensor developed to measure the non-nutritive sucking (NNS) performance and predict the nutritive sucking status in preterm infants.

**Methods:**

Preterm infants who were referred for feeding difficulty during the transition period from tubal feeding to oral feeding were enrolled in our study. A flexible pressure sensor was used to measure the non-nutritive sucking parameters of neonates. The evaluator stimulated the infants' lips and tongue with a pacifier integrated with a sucking pressure sensor, to check whether non-nutritive sucking had occurred. When the sucking reflex was induced, it was measured. The infants' sucking characteristics were subdivided into classifications according to the NOMAS criteria and full oral feeding (FOF) status. Quantitative NNS measurement according to the feeding state was compared between groups.

**Results:**

When comparing the quantitative NNS measurement by feeding characteristics, the average sucking pressure was significantly higher in infants in the FOF capable group than those in the incomplete FOF group. In addition, the maximum and average sucking pressure was significantly higher in infants with a normal sucking pattern compared to those with a disorganized sucking pattern. The average NNS pressure was divided over the range of 0–3 kPa and the same weight was assigned to each item. When the optimal cut-off value for the sensitivity and specificity of the average NNS pressure to estimate the FOF was set, a pressure of 1.5 kPa yielded the highest sensitivity (84.62%) and specificity (67.65%) on the receiver operating characteristic (ROC) curve. The area under the curve (AUC) was 0.786, and this result was statistically significant.

**Conclusions:**

This study presents a quantitative parameter for non-nutritive sucking in preterm infants with the use of a flexible pressure sensor. Results show possible quantitative indicators that can aid in predicting when preterm infants can transition to oral feeding and their prognosis. This will serve as a basis for future research on determining the feeding transition period of newborns with health conditions that affect oral feeding.

## Introduction

Oral feeding in newborns is considered safe and successful when the risk of aspiration is low; a given amount is ingested over an appropriate time while maintaining a stable cardiorespiratory status; and the sucking, swallowing, and breathing rhythms are harmonized (1). Oral feeding through breast milk or a bottle is the first developmental task of infants, and it is a necessary condition for discharge for infants admitted to the neonatal intensive care unit. Oral feeding is considered an innate ability, but it is nevertheless a very complex physiological process, especially in premature infants who are often unable to initiate oral feeding early in life (2). Due to the delayed start of oral feeding, nutrition needs to be taken through a tube for a long period, which may cause swelling of the laryngopharynx, sensory problems, gastroesophageal reflux, and increase the length of hospital stay, leading to a psychological and economic burden on the family (3, 4). Several prior studies have reported on factors predicting oral feeding readiness and strategies to promote oral feeding (5, 6). Oral feeding readiness can be defined in two contexts; first, when an infant who was previously maintained on gavage-tube feeding is first ready to start breastfeeding or bottle-feeding, and the infant is ready to participate in feeding; the latter indicates an infant's level of consciousness, physical condition, and expression of hunger. In addition, for oral feeding, the four hierarchical systems of autonomic, motor, behavior, attention, and interaction must be developed and integrated, making it very difficult to predict the preparation for oral feeding (7).

If oral feeding is delayed, the swallowing function of the infant can be checked by performing a videofluoroscopic swallow study or an endoscopic swallowing test. However, both tests focus on anatomical abnormalities, making it difficult to analyze functions quantitatively. Furthermore, neither reflects the impact of environmental factors. Although researchers have reached a consensus on the importance of oral feeding in newborns, quantitative data on the functional status that can determine the timing of the transition from tube to oral feeding and tube weaning are still absent (8). Non-nutritive sucking (NNS) is a predictable and rhythmic primitive reflex that is a precursor skill to oral feeding (9). Most previous studies predicted the oral feeding transition through quantitative analysis of the NNS (10, 11), Recently, a device for both assessment of NNS parameters and therapy through patterned and frequency-modulated oral stimulation with therapeutic pulses to train the infant's NNS skills was developed (12, 13). In addition, there was a contact-less method of quantifying NNS was developed by using video-based analysis of facial gesture. Most of the previous studies, however, were designed to include sensors, tubes or electronic components inside the pacifier mouthpiece, raising potential safety and hygiene issues (11, 14, 15). Although the common pressure range of NNS can be covered, they perform with low accuracy or resolution below the levels necessary to measure the sucking power of weaker infants. Previously, we reported the development of a new sensing device to measure infant's NNS pressure. The sensor was designed to work based on the strain gauge principle, and fabricated based on silver nanowires deposited on polydimethylsiloxane (PDMS) in a sandwich-like structure (16). This study thus aims to examine the relationship between the parameters of a silver nanowire-based flexible pressure sensor developed to measure the NNS performance, and the nutritive sucking status in preterm infants.

## Materials and methods

### Participants

This study enrolled 58 premature infants who were born between 25 and 36 weeks' gestational age (GA) and were referred to the division of pediatric rehabilitation for feeding difficulty during the transition period from tubal feeding to oral feeding. Infants were eligible for inclusion if (1) breathing was stable in room air, and (2) breathing assistance was not provided (breathing assistance was not considered to have occurred if a nasal cannula is used alone for low flow oxygen supply). Participants were excluded if they were not able to perform NNS (*n* = 9) or had orofacial malformations (*n* = 1) or unstable hemodynamic signs (including desaturation during NS) (*n* = 17) at the time of evaluation. A total of 31 preterm infants were enrolled and evaluated based on the inclusion and exclusion criteria.

### Test device for quantitative NNS measurement

**Figure 1:**
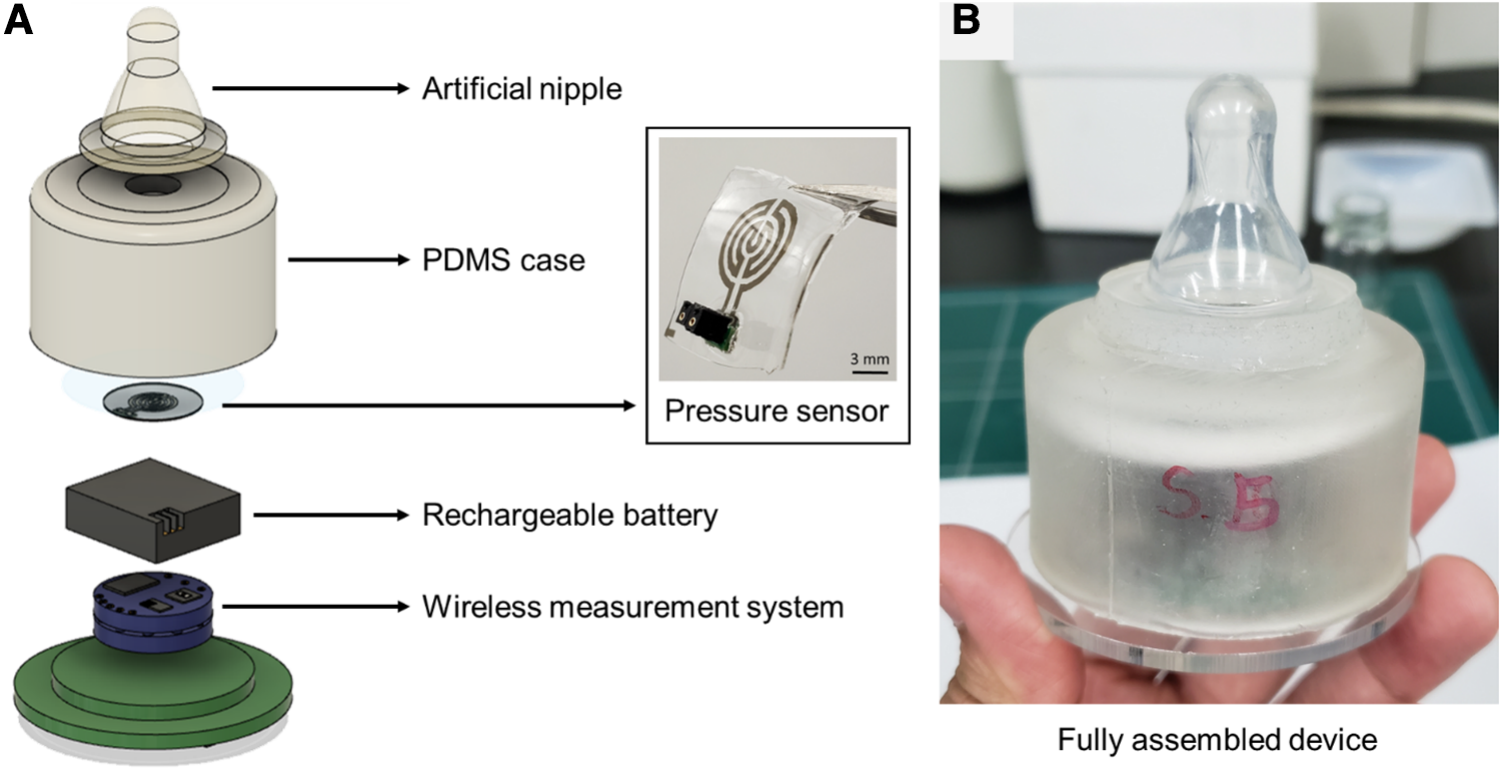
(**A**) Flexible pressure sensor and components comprising the wireless device to measure sucking pressure wirelessly, and (**B**) fully assembled device integrated with a commercial pacifier.

A flexible pressure sensor was used to measure the non-nutritive sucking parameters of neonates (Figure 1). The sensor was fabricated using silver nanowires deposited on PDMS in a sandwich-like structure. The pressure sensor was designed to measure pressure ranging from 0.15 kPa to 8 kPa, considering the range of sucking pressure of infants reported in previous studies (15, 17–19). The detailed processes to fabricate the sensor and the method to characterize it are described in our previous study (16). The sensor based on the principle of strain gauge was attached to a ring-shaped connecting module, and then to a pacifier. The negative sucking pressure exerted by the infant deformed the sensor membrane, causing its electrical resistance to change without creating any contact between the infant's mouth and the sensing element. The sensor weighed 0.4 g without the connecting module, and 3 g with it. The resultant thickness of the sensor was 440 μm. Using the developed sensor, no parts would be put inside the infant's mouth, or be in contact with the pacifier during the sensing of NNS power, which is fundamental to assuring the infant's safety and preserving the natural sucking behavior.

**Figure 2:**
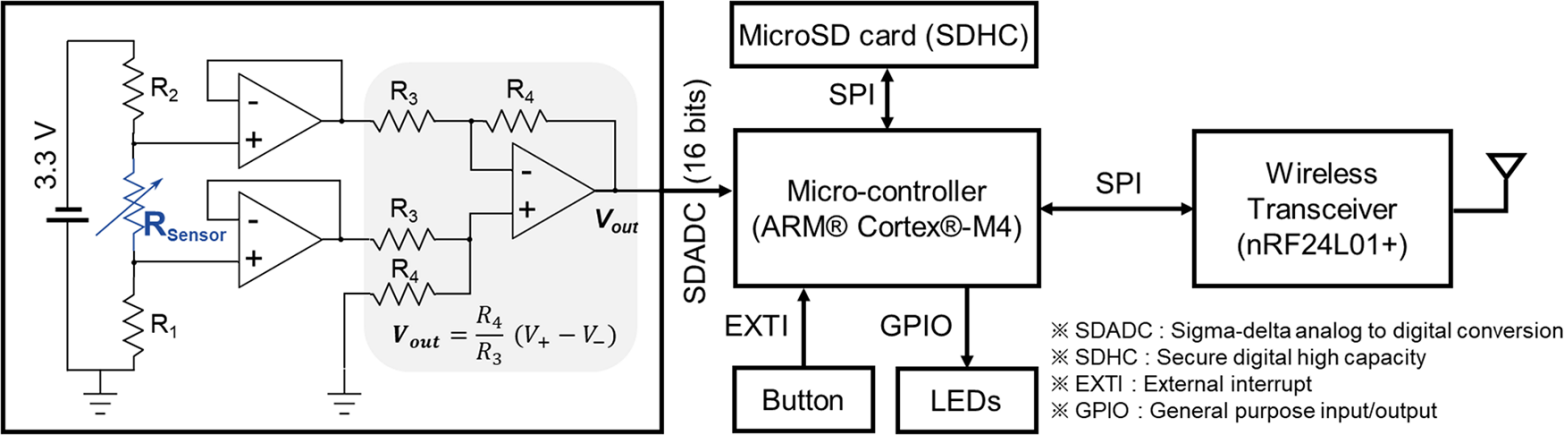
Circuit diagram of the wireless measurement system.

In preliminary experiments, we observed the distortion of signal when the sensor was connected to the instrument through long connection lines during measurement, which caused artifacts by unwanted movements of the lines. In addition, such a wired measurement environment limits the range of motion of the evaluator. To solve these problems, we developed a system that can measure the sucking pressure wirelessly (Figure 2). The measured data were transmitted in the 2.4 GHz Industry-Science-Medical band, and the data were collected at a sampling rate of 20 Hz. The transferred data were received and plotted in real-time on a laptop. At the same time, the data were saved in an SD card built into the device to prevent data loss by unexpected transmission errors. Using this wireless system, it was possible to collect more accurate data by removing the artifacts caused by the movement of the connection lines, which also freed the evaluator from the bounded working area.

### Feeding assessment

Full oral feeding (FOF), defined as oral feeding of more than 100 ml per kg per day without tube feeding for more than 48 h, was checked based on the evaluation time. A video was recorded to score the Neonatal Oral-Motor Assessment Scale (NOMAS) (20, 21), and this was independently evaluated by two NOMAS-certified rehabilitation medicine specialists. The video was recorded for 2 min from the time the sucking motion started in the lateral view so that the mouth, jaw, and neck motions could be clearly seen. Sucking difficulties were categorized into normal, disorganized, and dysfunctional sucking patterns according to the classification of the original NOMAS version (20). Most of measurements were obtained once per participant. As repetitive measurements affect the results due to fatigue, the correlation with oral feeding status was identified by measuring NNS immediately before the NOMAS assessment. The measurement was repeated on a different day for two participants as they did not show noticeable sucking behavior during the first measurement. One dataset per participant was used in the data processing and statistical analysis. The infants' sucking characteristics were subdivided into classifications according to the NOMAS criteria and FOF status. Within the subclassifications, the quantitative NNS parameter related to oral feeding status was identified by determining the difference in NNS measurements between the two groups. Quantitative NNS was measured immediately before the NOMAS assessment to determine its correlation with oral feeding status.

### Signal analysis of sucking responses

The changes in voltage detected by the sensor in response to sucking behaviors of the infants were converted into actual pressure values through post-processing. The first signal for a few seconds when there was no sucking response yet was taken as the baseline (V_base_), and the measured voltage value (V_meas_) was converted into the relative change in voltage (V_rel_). To correct the data caused by noise and disturbance, flattening was performed for each peak in the raw data by making the endpoint of the falling edge parallel to the starting point of the rising edge. The following equation was used to obtain V_rel_:

$$V_{\mathrm{rel}}\;=\;\frac{V_{\mathrm{meas}}-V_{\mathrm{base}}}{V_{\mathrm{base}}}$$

**Figure 3:**
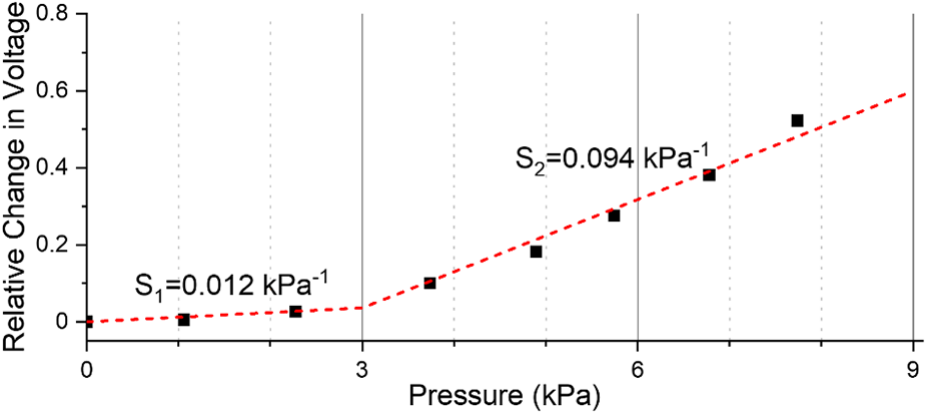
Representative example of the characteristic curve of a flexible pressure sensor.

Using the pressure characteristic curve for each sensor obtained by linear curve fitting (as shown in Figure 3), V_rel_ was converted into actual pressure values.

A sucking response was defined in the section where a significant change occurred from the baseline and was cross-checked with the synchronized video taken during the experiment. During the analysis, a phenomenon was observed in which a peak with a strong intensity and a peak with a weaker intensity were sequentially measured for a single sucking response. This was caused by consecutive responses in which positive pressure during the sucking motion with the deformation of the sensor, and then negative pressure was generated in returning the sensor to its original state in the subsequent exhalation. Since the sensor used in this experiment could not distinguish between positive and negative pressure, this phenomenon was detected as two consecutive strong and weak positive pressure peaks. Therefore, such paired peaks were treated as a single sucking response only for a strong peak. The following weak peak was regarded as an artifact and excluded from the analysis.

A suck burst was defined as a case in which sucking responses occurred continuously at two times per second in a non-nutritive sucking status. The duration of a suck burst was defined as the interval between the rising edge of the first peak of the burst and the falling edge of the last peak. The peak pressure could not be calculated in a few cases since the measured voltage was out of the operating voltage range. Nevertheless, since the voltage change pattern was observable, the remaining parameters except for the pressure value were calculated and used for behavior analysis. Based on those definitions, the number of sucking per unit time, the maximum and average pressure of sucking reflexes, the average number of sucking in the burst, and the maximum and average duration of sucking in the burst were calculated.

### Statistical analysis

Data were presented as means and standard deviations. The consistency between the evaluators on whether there was a stress sign during NOMAS finding and feeding was checked using the Cronbach α quantitative NNS measurement according to the feeding state and compared between groups using the Mann–Whitney *U* test. Sensitivity and specificity were assessed using the cut-off point on the average sucking pressure. The area under the receiver operating characteristic (ROC) curves (AUC) was derived, and the cut-off values were determined to predict the capability of FOF of the infants. *p* < 0.05 was considered statistically significant. All statistical analyses were estimated using SPSS version 25.0 (IBM SPSS, Armonk, NY, USA).

## Ethics statement

The study was approved by the Institutional Review Board (IRB) of Pusan National University Hospital (IRB No. 2012-031-098).

## Results

**Figure 4:**
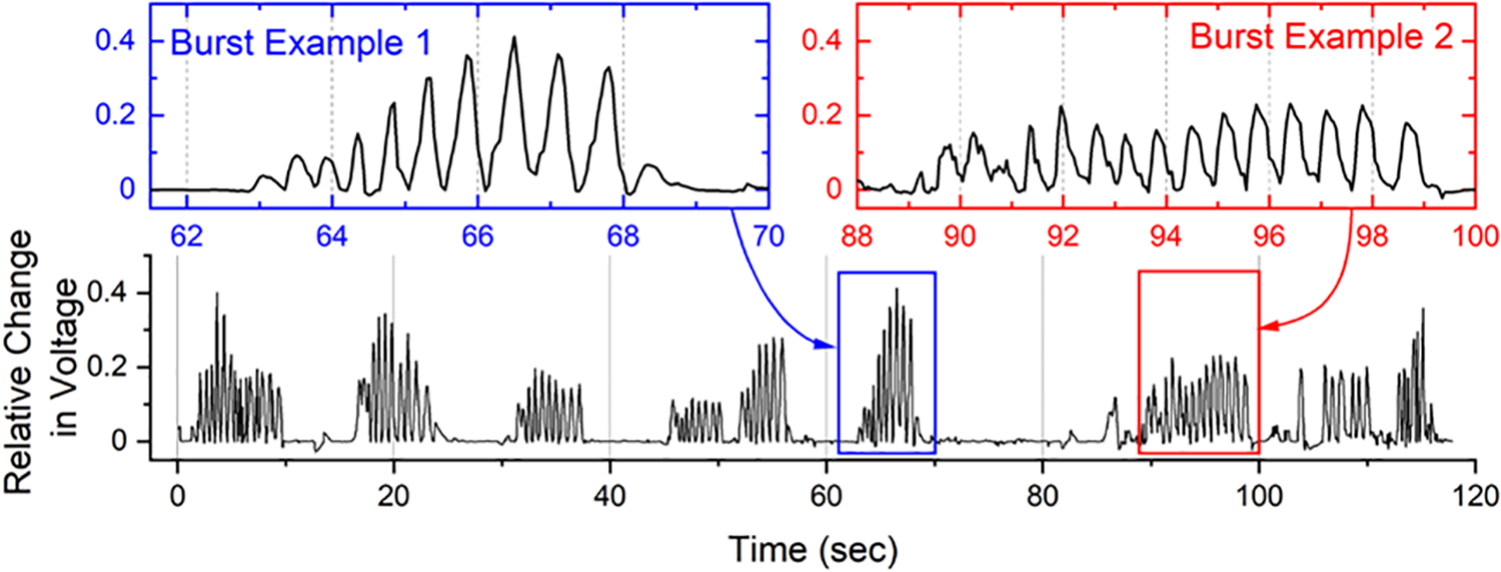
Representative example of the measured sucking pressure of a neonate. Bursts can be clearly identified.

All sensors were characterized prior to measuring the sucking response. Figure 3 shows the characterization result for a representative sensor. The characteristic curve was obtained by dividing it into two pressure ranges from 0 kPa to 3 kPa and from 3 kPa to 9 kPa. For the sensor shown in Figure 3, the sensitivity in the pressure range of 0 kPa–3 kPa was 0.012 kPa^−1^, and the sensitivity in the pressure range above 3 kPa was 0.094 kPa^−1^. A representative example of the measured sucking pressure of a neonate is shown in Figure 4. Differences between baseline and sucking responses were evident, and several bursts were identified. The total number of sucking responses was 99, and the number of sucking responses per unit time was 50.4 times per minute. The maximum pressure was 8.37 kPa, and the average pressure was 3.95 ± 1.61 kPa. The total number of times that suck burst was induced was 10, and the average number of suck responses during suck burst was 9.8 ± 4.29. The maximum duration of the suck burst was 9.95 s, and the average duration was 5.61 ± 2.37 s.

A total of 31 preterm infants were enrolled and evaluated based on the inclusion and exclusion criteria. The baseline characteristics are shown in Table 1. In regard to NOMAS, 12 (38.7%) infants showed a normal pattern and 19 (61.3%) showed a disorganized pattern. There were no infants showing a dysfunctional sucking pattern. The interrater reliability for the NOMAS finding was Cohen's *κ* = 0.925, and the presence of the stress signs was higher with a Cohen's κ of 0.983.

**Table 1:** Clinical characteristics of the infants.

Characteristics	Total (*n* = 31)	Complete FOF (*n* = 23)	Incomplete FOF (*n* = 8)	*p*-value
Gestational age (wks)	33.03 ± 3.16	33.52 ± 2.71	31.62 ± 4.10	0.263
Sex (Male/Female)	18/13	13/10	5/3	0.772
Birthweight (g)	1,907.68 ± 593.10	1,997.69 ± 578.13	1,648.87 ± 594.92	0.190
Apgar score (5 min)	7.38 ± 1.35	7.43 ± 1.34	7.25 ± 1.48	0.726
Surfactant use	16 (51.6%)	12 (52.1%)	4 (51.6%)	0.917
Caffeine therapy for AOP	5 (16.1%)	3 (13.0%)	2 (51.6%)	0.436
Intubation history	18 (58.0%)	12 (52.1%)	6 (75.0%)	0.268
Hx. Of respiratory assist	23 (74.1%)	16 (69.5%)	7 (87.5%)	0.326
Nasal O2	23 (74.1%)	16 (69.5%)	7 (87.5%)	0.239
Hood O2	9 (29.0%)	7 (30.4%)	2 (25.0%)	0.774
HFNC (3L/kg)	22 (70.9%)	15 (65.2%)	7 (87.5%)	0.239
CPAP	7 (22.5%)	2 (8.6%)	5 (62.5%)	0.002*
Mechanical ventilation	18 (58.0%)	12 (52.1%)	6 (75.0%)	0.268
RDS	16 (51.6%)	12 (52.1%)	4 (50.0%)	0.917
BPD	8 (25.8%)	7 (30.4%)	1 (12.5%)	0.758
GMH or IVH on brain ultrasonography	6 (19.3%)	4 (17.3%)	2 (25.0%)	0.644
Corrected age at evaluation (wks)	36.03 ± 1.55	36.00 ± 1.47	36.12 ± 1.88	0.926

Continuous variables following the normal distribution are denoted by mean ± SD, Categorical variables are denoted by *n* (%).

FOF, full oral feeding; AOP, apnea of prematurity; HFNC, high flow nasal cannula; CPAP, continuous positive airway pressure; RDS, respiratory distress syndrome; BPD, bronchopulmonary dysplasia; GMH, germinal matrix hemorrhage; IVH intraventricular hemorrhage.

*Significant difference (*p*<0.05).

The feeding characteristics of infants at the time of evaluation are shown in Table 2. According to NOMAS, the group with the normal sucking pattern sustained significantly longer sucking per time and the presence of the sucking and rooting reflexes were significantly higher in the normal group compared to the disorganized group. The NNS characteristics for the infant divided by FOF status and NOMAS finding is shown in Table 3. When comparing the quantitative NNS measurement by feeding characteristics, the average sucking pressure was significantly higher in the FOF-capable group than in the incomplete-FOF group. In addition, the maximum and average sucking pressures were significantly higher in infants with a normal sucking pattern when compared to those with a disorganized sucking pattern (Table 3).

**Table 2:** Comparison of feeding characteristics of the infants by NOMAS finding.

Characteristics	Total (*n* = 31) (%)	Normal (*n* = 12) (%)	Disorganized (*n* = 19) (%)	*p*-value
Amount of feeding per feeding (cc/time)	42.90 ± 11.82	46.50 ± 12.70	41.2 ± 11.30	0.831
Amount of feeding per day (cc/day)	343.35 ± 94.69	372.00 ± 101.63	329.71 ± 94.69	0.723
Duration of feeding per time (minute/time)	16.00 ± 3.94	23.33 ± 6.39	20.96 ± 6.63	0.003*
Presence of sucking reflex	17 (54.5%)	9 (90%)	8 (38.0%)	0.008*
Presence of rooting reflex	16 (51.6%)	9 90%)	7 (33.3%)	0.004*

NOMAS, neonatal oral-motor assessment scale.

*Significant difference (*p*<0.05).

**Table 3:** Comparison of quantitative NNS measurement by feeding characteristics.

(a) Comparison of quantitative NNS measurement by FOF status			
Characteristics	Complete FOF (*n* = 23)	Incomplete FOF (*n* = 8)	*p*-value
Total number of sucks	61.25 ± 30.30	56.00 ± 43.68	0.484
Average number of sucks per minute (times/min)	19.61 ± 9.67	25.01 ± 23.01	0.842
Maximum pressure of suck (kPa)	4.14 ± 2.25	2.87 ± 2.20	0.222
Maximum pressure of suck (cmH_2_O)	42.4 ± 23.0	29.4 ± 22.5	
Average pressure of suck (kPa)	2.22 ± 1.07	1.12 ± 0.51	0.033*
Average pressure of suck (cmH_2_O)	22.7 ± 11.0	11.5 ± 5.22	
Total number of suck bursts	11.87 ± 10.82	10.82 ± 7.13	0.550
Average number of sucks per burst	4.03 ± 1.35	3.85 ± 2.06	0.411
Maximum length of bursts (sec)	5.25 ± 3.21	4.59 ± 2.52	0.580
Average length of bursts (sec)	2.32 ± 0.99	2.34 ± 1.06	0.877
(b) Comparison of quantitative NNS measurement by NOMAS finding			
Characteristics	Normal (*n* = 12)	Disorganized (*n* = 19)	*p*-value
Total number of sucks	67.16 ± 47.45	51.15 ± 34.98	0.389
Average number of sucks per minute (times/min)	33.13 ± 27.30	17.61 ± 11.80	0.120
Maximum pressure of suck (kPa)	5.53 ± 2.04	2.83 ± 1.66	0.046*
Maximum pressure of suck (cmH_2_O)	56.5 ± 20.9	28.9 ± 17.0	
Average pressure of suck (kPa)	2.83 ± 1.15	1.66 ± 0.89	0.046*
Average pressure of suck (cmH_2_O)	28.9 ± 11.8	17.0 ± 9.10	
Total number of suck bursts	10.58 ± 6.50	11.42 ± 7.13	0.921
Average number of sucks per burst	4.66 ± 2.53	3.41 ± 1.15	0.252
Maximum length of bursts (sec)	5.17 ± 3.07	4.50 ± 2.45	0.589
Average length of bursts (sec)	2.56 ± 1.40	2.19 ± 0.71	0.734

*Significant difference (*p*<0.05).

The average NNS pressure was divided over the range of 0–3 kPa and the same weight was assigned to each item. When the optimal cut-off value for the sensitivity and specificity of the average NNS pressure to estimate the FOF was set, a pressure of 1.5 kPa yielded the highest sensitivity (84.62%) and specificity (67.65%) on the ROC curve. The AUC was 0.786, and this result was statistically significant (Table 4).

**Table 4:** Criterion values of average sucking pressure and coordinates of the ROC curve.

Cut off value of average sucking pressure (kPa)	Sensitivity	95% CI	Specificity	95% CI
0.00	100	75.3–100.0	0	0.0–10.3
0.5	100	75.3–100.0	0	0.0–10.3
1.00	100	75.3–100.0	26.47	12.9–44.4
1.50	84.62	54.6–98.1	67.65	49.5–82.6
2.00	46.15	19.2–74.9	94.12	80.3–99.3
2.50	7.69	0.2–36.0	100	89.7–100.0
3.00	0	0.0–24.7	100	89.7–100.0

## Discussion

Oral feeding is one of the most important skills developed during infancy. Improper development of this skill can lead to not only nutritional consequences, but also physical concerns, such as aspiration pneumonia and financial difficulties. Therefore, indicators that objectively evaluate oral feeding readiness are needed. Although quantitative indicators that assess physiologic function are clinically meaningful, there is a need for an objective evaluation system because the experiences and standards of clinicians in quantitatively measuring sucking function and presenting guidelines for safe oral feeding are diverse (20, 22).

Prior researchers have attempted to develop a device that checks nutritive and non-nutritive sucking performance (14, 17). These devices did not have an electrical connection to the pacifier itself and instead made measurements remotely through a connected pressure transducer. Furthermore, due to the weight of the sensor, there was a limitation in carrying out this test on infants with very weak sucking power. Previously a flexible pressure sensor based on the strain gauge principle was designed and fabricated to measure the non-nutritive sucking power of infants (16). The sensor was optimized to achieve both suitable sensitivity and stability. With this device of excellent long-term electro-mechanical stability and high sensitivity, the current study was able to present quantitative NNS parameters for preterm infants and was also able to show significant results related to infant feeding and function.

Intact NNS is a skill of infancy that is important for determining oral feeding readiness and full oral feeding. Although the relationship between NNS and NS is not conclusive, previous studies that mostly evaluated sucking performance by observation do not suggest any correlation or cut-off point of objective sucking parameters suitable for the implementation of oral feeding (23, 24). A complete suck-swallow-breathe cycle is necessary to maintain physiologic stability during feeding, and since the suck-swallow-breathe synchrony is fully developed after 34 weeks of gestation, effective feeding may not be achieved in premature infants.

In particular, NNS has a significant effect on the transitions from gavage to full oral feeding and from the start of oral feeding to full oral feeding, as well as the length of hospital stay, reflecting the neurobehavioral maturation and organization of the infant (25).

The most notable result was the sucking pressure in NNS, which, among the quantitative indicators related to various NNS performance, had a significant correlation with FOF and the mature sucking pattern. NNS has a stereotypical pattern, with an average of 2 sucks per second, occurring between 6 and 12 times per burst (26). Therefore, the formation of a regular and consistent NNS pattern is the precursor to the development of the oral feeding skill (27). Previous studies showed that well-developed newborns induced nearly 15 bursts of NNS in 5 min on average, but the sucking amplitude according to the bursts was not constant (28). However, sufficient feeding cannot be continued over time with bottle-feeding premature infants because of the incoordination of the suck-swallow-breathe cycle and fatigue. Even if sucking performance is induced, not enough sucking pressure may be formed to cause an appropriate negative pressure for the milk to pass through the pacifier. Hence, as the results demonstrate, determining whether there is enough sucking power during NNS to allow for significant bottle feeding is more meaningful than relying on the duration and number of sucks or bursts to predict oral feeding development.

The normal range of sucking power for proper oral feeding in preterm infants has not been suggested in previous studies. Our study shows that when estimating FOF capability, an NNS pressure of 1.5 kPa yielded the highest sensitivity and adequate specificity on the ROC curve. Quantitative indicators like this will be useful when determining the appropriate time to attempt safe oral feeding.

There are several limitations to our study. First, the number of participants in the final enrollment was relatively small. To apply this device in practice, the validation of the parameters for a larger number of patients should be conducted. Second, due to the limited number of participants, a detailed analysis of the range of the GA of preterm children could not be conducted. Going forward, it will be clinically crucial to confirm any changes in the quantitative parameters for newborns before and after 34 weeks GA, which is the time when the suck-swallow-breathe synchrony is fully coordinated. The confirmation of changes in the NNS parameters for newborns in early preterm and late preterm is clinically important. Lastly, NS performance was not assessed in this study. Therefore, oral feeding compliance, significant indicators, and cut-off points were presented as NNS parameters, and the associations with feeding status were evaluated in this study.

## Conclusion

This study used a wireless, flexible pressure sensor to present a quantitative parameter for NNS in preterm infants. Results suggest quantitative indicators that can help determine when to transition these infants to oral feeding and also predict their prognosis. These results will serve as a basis for future research on the transitional feeding of newborns who have health conditions that affect oral feeding.

## Data Availability

The original contributions presented in the study are included in the article. Further inquiries can be directed to the corresponding author.
